# Culture and Maintenance of Human Embryonic Stem Cells  

**DOI:** 10.3791/1427

**Published:** 2009-12-22

**Authors:** Lia Kent

**Affiliations:** Research and Development, Stemgent

## Abstract

Human embryonic stem (hES) cells must be monitored and cared for in order to maintain healthy, undifferentiated cultures. At minimum, the cultures must be  fed  every day by performing a complete medium change to replenish lost nutrients and to keep the cultures free of unwanted differentiation factors. Although a small amount of differentiation is normal and expected in stem cell cultures, the culture should be routinely cleaned up by manually removing, or "picking" differentiated areas. Identifying and removing excess differentiation from hES cell cultures are essential techniques in the maintenance of a healthy population of cells.

**Figure:**
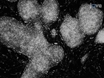


## Protocol

### 1. Maintenance: Observing Cultures Under the Microscope

1. Remove one plate of hES cells from the 37°C incubator and bring to the microscope.
2. Observe the colonies at low power, using 2X or 5X magnification, to assess the overall culture quality. Note the following: normal medium color, absence of any visible contamination, overall colony size, quality and density, and any other observations.
3. Observe medium color changes. Due to the change in pH and the depletion of nutrients in the medium after an overnight incubation with the hES cells, the medium will change from a red hue to a "straw" color. If the medium appears purple, a basic pH shift has occurred. If the medium appears extremely yellow, the medium has acidified. Very yellow medium may be the result of extremely overcrowded colonies in the well or contamination. The medium should appear clear at all times. Though a certain level of cell debris in the medium is normal, it should never appear cloudy. If a high level of cell debris is observed, the culture is not healthy and may be contaminated.
4. If the cultures do not require maintenance or passaging, they can be taken to the biological safety cabinet to be fed (see section 2).
5. If the cultures contain more than approximately 10% differentiating colonies, the cells should be "cleaned up" by manually removing the differentiated areas (see section 3).
6. If the cultures contain large or dense colonies, or the MEF feeder layer is more than 12 days old, the cells should be passaged (see section 4).

### 2. Feeding hES Cells

1. In a sterile biological safety cabinet, remove the lid of the culture dish and aspirate the spent medium. Do not allow the tip of the pipette to touch any part of the plate other than directly inside the wells. If there is any concern of contamination, change to a new, sterile pipette.
2. Using a sterile glass pipet, add the appropriate amount of warmed hES cell culture medium to each well. For cultures in a 6-well plate, add 2.5 mL medium per well.
3. Return the plate to the incubator to remain at 37°C and 5% CO_2_.

### 3. Removing Differentiation from hES Cell Cultures

1. Transform 9 inch glass Pasteur pipettes into picking tools by molding the tips over the controlled flame of an alcohol burner. Be sure that the tip of the pipette is sealed to prevent contamination and rounded to avoid scratching the plastic of the culture dish. Sterilize the picking tools before use using the UV light source in the biological safety cabinet or picking enclosure.
2. Remove the differentiated areas. Differentiation often occurs in only a portion of an hES cell colony, usually along the edges or as isolated spots in the center. If only a section of a colony is differentiating, only the differentiated area needs to be removed. Differentiation can often lift off the plate in a "sticky" sheet, and it is helpful to first separate the differentiated cells from the rest of the colony by drawing a line through the colony with the end of the picking tool.
3. Once the pieces of the colony are separated, gently glide the picking tool along the plate to detach the unwanted cells. Take care not to scrape away too much of the MEF feeder layer between the colonies in this process.
4. When all of the differentiated cells are removed from the plate (and floating in the medium), return the culture to the biological safety cabinet. Aspirate the medium containing the differentiated cells and replace with fresh hES cell culture medium.

### 4. Passaging hES Cells

#### Enzyme Incubation

1. In a sterile biological safety cabinet, remove the lid of the 6-well culture dish and aspirate the spent medium from the wells to be passaged. Do not allow the tip of the pipette to touch any part of the plate other than directly inside the wells.
2. Using a sterile glass pipet, add 1 mL of warmed collagenase IV to each well to be passaged.
3. Return the plate to the 37° C incubator for 5 minutes.

#### Scraping and Pooling the hES Cells

1. After incubating the cells with collagenase, observe the colonies under the microscope. The enzyme should cause a subtle but observable change in the colony edges.
2. In the biological safety cabinet, gently aspirate the enzyme from each well and replace with 2.0 mL hES cell culture medium.
3. Tip the culture plate slightly toward you and take up the 2.0 mL medium in the first well with a 5 mL glass pipet. Holding the pipet perpendicular to the bottom of the plate, gently glide the pipet tip across the well while slowly releasing the medium.
4. Repeat the scraping and pipetting motion 3-4 times until all of the colonies have been removed from the well. Pipet gently to avoid breaking up the colonies into too small of pieces. When the cells have been removed from the first well, leave the contents in the well and begin scraping the cells off of the next well.
5. When all of the wells to be passaged have been scraped, pool the medium containing the colony pieces from each well in a sterile 15 mL conical tube.
6. Rinse the scraped wells by adding 1 mL of hES cell culture medium to each well. Collect this rinse and transfer to the conical tube.
7. Pipet the cells gently in the tube to break up the colony pieces up to the desired size.
8. Centrifuge the cells for 5 minutes at 200 x g.

#### Plating the hES Cell Cells

1. While the hES cells are in the centrifuge, prepare a new 6-well MEF feeder plate. Label the plate with the appropriate hES cell information: cell line, new passage number, and passage date. Aspirate the MEF medium from the wells and add 1.0 mL PBS to each well. Gently swirl the buffer around the wells and aspirate the PBS wash. Add 1.5 mL hES cell culture medium to each well and set the plate aside.
2. When the hES cells are finished centrifuging, bring the tube back to the biological safety cabinet. Aspirate the supernatant, being careful not to disturb the loosely-packed cell pellet.
3. Resuspend the cells in the pellet with enough medium for 1 mL hES cell culture medium per well to be plated. When splitting into 6 new wells, resuspend the pellet in 6 mL medium.
4. Gently and evenly add 1 mL of the cell suspension to each prepared well of the MEF feeder plate.
5. Place the plate into the 37°C incubator and carefully slide the plate forward to back, and side to side to evenly distribute the cells throughout the well.
6. Allow the cells to attach at 37°C overnight.

### 5. Representative Results:

Undifferentiated hES cells are small, tightly-packed, and usually consist of larger, more spread out cells. Differentiation can occur within a colony (Figure 1A) or between colonies (Figure 3B).

Different morhopologies can be seen under low magnification under the microscope. A good cell morphology, as seen in Figure 2A, contains small, tightly packed cells that grow in a monolayer. Cells should have clean, defined edges, with little to no differentiation. The cells shown in Figure 2B are ready for passaging, and the cells shown in the Figure 2C are overcrowded.


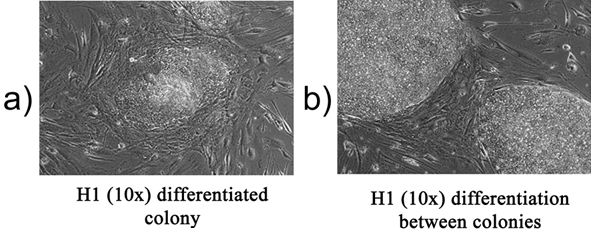
 **Figure 1. Differentiation in hES cultures.** (A) differentiation of a colony (B)differentiation between colonies.


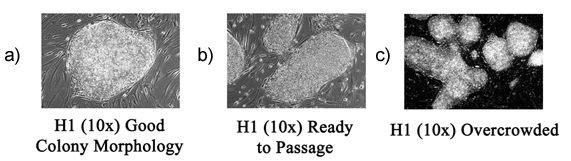
 **Figure 2. Morphologies seen in hES cultures**. (A)good cell morphology (B) hES cells that are ready for passaging (C) overcrowded cells.

## Discussion

Sterility must be maintained at all times when working with hES cells. Clean and sterilize the biological safety cabinet and all equipment before use. All reagents must be filtered using a 0.22 μm pore size filter prior to use. The use of antiboiotics in hES cell culture is not necessary and should be avoided.

A separate environment should be set up as a designated picking station. The sterile enclosure for the station can be a static enclosure (such as a PCR workstation) with a UV light source, a laminar flow hood, or a biological safety cabinet. A dissecting microscope inside the picking station is needed to observe the colonies as the differentiated areas are removed. The front glass panel of a static enclosure or a biological safety cabinet must be modified to allow for the oculars of the microscope to extend through the panel without compromising proper airflow and sterility.

To maintain healthy hES cell cultures, cells must be passaged at the optimal time, typically every 4-7 days. At this time the colonies have reached their maximum size and may be beginning to merge. Merged colonies can increase the rate of differentiation in the culture. Split ratios generally fall between 1:3 and 1:5, depending on the number of colonies plated, expansion rate, and culture conditions. Overplated colonies (split ratio too low) will likely merge prematurely and need to be passaged before they reach their maximum size.

Cultures on an MEF feeder layer that is more than 2 weeks old must be passaged on to new MEFs that will support undifferentiated growth and proliferation.

Since differentiation naturally occurs in hES cell cultures, a small amount is expected. Frequent or excessive differentiation may occur if the cultures do not receive appropriate care. The cells must be fed every day, overgrowth between passages should be avoided. Always use fresh reagents. All of the media, MEFs and reagents should be used within 14 days to avoid undifferentiated hES cell growth. New lots of reagents, such as Knockout Serum Replacement, FBS and MEFs, should be screened prior to use. Remember that any differentiated cells not removed prior to passaging will be transferred to the new cultures. Also, try to keep the cells at 37°C whenever possible. Minimize the time the cells spend outside of the incubator (e.g. on the microscope stage or in the biological safety cabinet.) A heated microscope stage can be very beneficial if the cells will be of the incubator for extended periods of time.

When observing the hES cell cultures under the microscope, first assess the overall quality and size of the colonies view using a low power (2x or 5x) objective. If potentially differentiated cells are observed at low power, confirm the differentiated morphology using a higher magnification objective (10X).

Immediately after removing differentiation, the edges of the colonies may appear jagged or damaged. Incubate the colonies overnight in fresh medium to allow the remaining undifferentiated cells to recover. Without the influence of differentiation in the culture, these remaining colonies will continue to proliferate normally.

If possible, begin experiments using low-passage hES cells. Karyotype the cells before and after all major experiments.
